# Short-course letrozole restores fertility in aged azoospermic dogs

**DOI:** 10.3389/fvets.2026.1939063

**Published:** 2026-10-09

**Authors:** Bao Liu, Lei Zhang, Fangzhou Zhu, Xin Jin, Taole Liu

**Affiliations:** 1 Suzhou Biosynium Corporation, Suzhou, Jiangsu, China; 2 School of Public Health, Suzhou Medical College, Soochow University, Suzhou, Jiangsu, China; 3 School of Basic Medical Sciences, Suzhou Medical College, Soochow University, Suzhou, Jiangsu, China

**Keywords:** aging, azoospermia, dog, fertility, letrozole, spermatogenesis, testosterone, theriogenology

## Abstract

**Introduction:**

Age-related azoospermia is a clinically important cause of male infertility in dogs, yet effective pharmacological treatments for aged males remain limited. Whether transient aromatase inhibition can restore fertility in aged azoospermic dogs without prolonged exposure is unclear.

**Methods:**

This study evaluated whether a 1-week course of letrozole, an aromatase inhibitor, could restore spermatogenesis and fertility in aged azoospermic male dogs. Untreated aged azoospermic and young fertile dogs served as comparison groups, and a separate cohort of five aged dogs received 3 months of letrozole for safety assessment. Serum testosterone, semen parameters, testicular histology, fertilization-related transcripts, spermatogonia, seminal plasma metabolites, and serum biochemical markers of liver, kidney, and bone metabolism were assessed before and after treatment over a 6-month follow-up.

**Results:**

Serum testosterone increased within 1 week and peaked at approximately 2 weeks. Spermatozoa reappeared in ejaculates approximately 3 months after treatment and persisted for more than 6 months; treated males subsequently achieved pregnancies with healthy offspring, although litter size remained smaller than that of young fertile dogs. Compared with 3-month administration, 1-week treatment was not associated with detectable changes in serum alkaline phosphatase (ALP).

**Discussion:**

These findings indicate that a 1-week letrozole course can restore spermatogenesis and fertility in aged azoospermic dogs, supporting its further evaluation as a low-exposure therapeutic strategy; larger, breed-stratified studies with extended safety profiling are recommended before clinical adoption.

## 1 Introduction

Male infertility affects both animals and humans and is a common problem in veterinary reproduction, reducing breeding efficiency in several species, including dogs (1). In dogs, infertility may result from genetic abnormalities, testicular disease, endocrine imbalance, reproductive tract disorders, or advanced age (2). Age-related infertility is particularly relevant in working dogs, breeding males, and companion animals for which preservation of reproductive capacity has practical and emotional value.

Azoospermia is defined as the complete absence of spermatozoa in the ejaculate and is a severe form of male infertility. In aged male dogs, azoospermia is often associated with degenerative testicular changes, including reduced seminiferous tubule diameter, fewer germ cells, and impaired Leydig cell function (2). Leydig cells synthesize and secrete testosterone, a key hormone that supports spermatogenesis, sexual behavior, and male secondary sexual characteristics (3). With aging, Leydig cell number and function decline, reducing testosterone availability and contributing to impaired spermatogenesis (1). Aromatase converts androgens, including testosterone, into estrogens (4) and is expressed in the testes, adipose tissue, brain, and other tissues, where local estrogen production participates in testicular regulation. Notably, aromatase activity and the aromatization of androgens to estrogens increase with age in males, contributing to a relative shift toward estrogen excess as testosterone declines (5).

Aromatase inhibitors such as letrozole reduce the conversion of testosterone to estrogens and can lessen estrogenic negative feedback on the hypothalamic–pituitary-gonadal (HPG) axis (6). This response may increase testosterone, follicle-stimulating hormone (FSH), and luteinizing hormone (LH), which together support Sertoli cell function, Leydig cell steroidogenesis, and germ-cell development (6–9). Letrozole has been evaluated for male infertility across several species, increasing testosterone and improving spermatogenesis or fertility in aged roosters (10), mice with experimentally induced infertility (11), markhoz bucks (12), rats (13), and dogs [(14); present study]. Among these preclinical studies, safety was assessed to varying degrees; for example, Mogheiseh et al. (14) evaluated prostate dimensions and endocrine balance in dogs, whereas most reports focused on efficacy endpoints and lacked comprehensive toxicity profiling. In males, estrogens are physiologically important for maintaining bone mineral density and for sperm maturation and survival within the epididymis; thus, aromatase inhibition and the resulting estrogen suppression may adversely affect skeletal and reproductive-tract health (15). However, current knowledge of letrozole effects in aged azoospermic male dogs remains limited. Consequently, potential concerns—including estrogen suppression, bone health, and prostate effects—may restrict the broader use of letrozole in older males (6, 14). Because aromatase inhibition lowers circulating estrogens, these safety concerns, particularly with prolonged exposure, warranted careful evaluation in the present study. Therefore, the present study aimed to determine whether a 1-week course of letrozole can restore endocrine status, spermatogenesis, semen quality, and fertility in aged azoospermic male dogs while limiting the safety concerns associated with longer exposure. To assess spermatogenic recovery, conserved fertilization-related transcripts (*IZUMO1*, *SPACA6*, and *TMEM95*) and the differentiating spermatogonia marker *KIT* (CD117) (27) were evaluated alongside semen parameters, testicular histology, seminal plasma metabolites, and selected serum biochemical indicators of liver, kidney, and bone metabolism.

## 2 Materials and methods

### 2.1 Ethical approval

The animal study was reviewed and approved by the Soochow University Animal Ethics and Use Committee (approval No. SUDA20260203A03). The study was conducted in accordance with local legislation and institutional requirements. Written informed consent was obtained from the owners for the participation of their animals.

### 2.2 Study design, location, and animal allocation

This prospective observational and interventional study was conducted at collaborating companion-animal clinics and breeding kennels in Suzhou, Jiangsu Province, China, with a 6-month follow-up period per enrolled dog. Dogs were allocated to groups on the basis of clinical presentation and the owner’s informed consent to participate: (i) a young fertile control group (*n* = 5); (ii) an aged azoospermic untreated control group (*n* = 3); (iii) an aged azoospermic treatment group that received 1 week of letrozole (*n* = 5); and (iv) an aged safety group that received 3 months of letrozole (*n* = 5). Group assignment was non-randomized and dictated by the clinical availability of aged azoospermic dogs and owner willingness to enroll; young fertile and untreated aged dogs served as reference and disease controls, respectively. The 1-week letrozole group was monitored for the full 6-month follow-up.

### 2.3 Animals, inclusion and exclusion criteria

Thirteen aged dogs (6–9 years) and five young dogs (2 years) were included. Detailed group composition is provided in Table 1. The aged azoospermic treatment group (*n* = 5) comprised one Golden Retriever, one Japanese Akita, one Border Collie, one Shiba Inu, and one Welsh Corgi. The untreated aged azoospermic control group (*n* = 3) comprised one Bichon Frise, one German Shepherd, and one Beagle. The 3-month letrozole safety group (*n* = 5) consisted of Beagle dogs (8–12 kg, 8 years). The young fertile control group (*n* = 5) comprised Beagle dogs (8–11 kg, 2 years).

**Table 1:** Key demographics, treatments, and procedures in the experimental and control groups.

Group	*n*	Dog characteristics	Treatment	Investigations
Young fertile	5	Healthy males (Beagle, 8–11 kg, 2 years old)	No treatment	Semen quality, hormone testing, seminiferous histology, fertility, and safety indicators
Aged azoospermic + 1-week letrozole	5	Azoospermic males (Golden Retriever, 30 kg, 6 years old; Akita, 35 kg, 7 years old; Border Collie, 18 kg, 8 years old; Shiba Inu, 10 kg, 9 years old; Corgi, 13 kg, 7 years old)	Letrozole for 1 week	Semen quality, hormone testing, seminiferous histology, fertility, and safety indicators
Aged azoospermic untreated	3	Azoospermic males (Bichon Frise, 5 kg, 8 years old; German Shepherd, 33 kg, 6 years old; Beagle, 10 kg, 8 years old)	Untreated control	Semen quality, hormone testing, seminiferous histology, fertility, and safety indicators
Aged + 3-month letrozole	5	Aged males (Beagle, 8–12 kg, 8 years old)	Letrozole for 3 months	Serum indicators related to bone, liver, and kidney safety

Inclusion criteria: clinical health without obvious systemic disease; azoospermia confirmed by weekly semen analyses over 2 months and documented for at least 1 year before study initiation; no history of reproductive tract trauma, orchitis, or urinary disease; no hormonal drug or aromatase inhibitor use within the previous 6 months; and, for aged dogs, documented successful mating ability and fertility earlier in life.

Exclusion criteria: abnormal hepatic or renal function; chronic systemic disease; reproductive tract inflammation; obvious testicular atrophy or lesions; or previous hormonal intervention.

Because aged spontaneously azoospermic dogs are a rare and heterogeneous clinical population, the available animals represented multiple breeds of differing body size and metabolic rate. This breed heterogeneity reflects recruitment constraints rather than a designed comparison and is acknowledged as a limitation (see Limitations).

### 2.4 Diagnosis of azoospermia and clinical examination

Azoospermia was diagnosed by a veterinarian (reproductive theriogenology) on the basis of the complete absence of spermatozoa in semen collections obtained by manual stimulation, supplemented by testicular palpation. All dogs additionally underwent a general physical examination performed by the same veterinarian. This examination included palpation of the testes and accessory sex glands and was used to exclude testicular or reproductive tract disorders before enrollment.

### 2.5 Animal care and monitoring

Dogs were housed under standard kennel conditions at the collaborating clinics and kennels, with free access to water and a balanced commercial diet; although environmental conditions were not experimentally standardized across sites, all animals received consistent basic husbandry. Throughout the study, all dogs received routine clinical monitoring at fixed time points, including weekly physical and behavioral examinations during the 1-week intervention and scheduled follow-up assessments until the 6-month endpoint. Potential adverse reactions were assessed by monitoring appetite, daily activity, behavior, and general physical condition.

### 2.6 Blood sample collection and serum preparation

Blood samples were collected from a cephalic or jugular vein after a 12 h overnight fast. For measurement of serum testosterone concentration, a single basal morning sample was collected within a fixed time window to minimize diurnal variation; additional samples were collected weekly during and after treatment as specified per group. For serum biochemistry, blood was collected 24 h after the final letrozole dose. Collected blood was allowed to clot at room temperature and then centrifuged at 3000 × g (RCF) for 10 min to obtain serum, which was aliquoted and stored at −80 °C until analysis. Hemolyzed or lipemic samples were excluded because free hemoglobin and lipids interfere with the spectrophotometric and enzymatic assays used for hormone and metabolite quantification, which would otherwise bias the results.

### 2.7 Testicular biopsy

Testicular biopsies were obtained from the young fertile, untreated aged azoospermic, and 1-week letrozole-treated groups at baseline (before treatment) and at specified follow-up time points to evaluate seminiferous tubular morphology and spermatogenic stage composition and to monitor morphological recovery. The 3-month safety group was not biopsied, as this cohort was established solely for serum biochemical safety profiling. Biopsies were performed under local anesthesia with lidocaine. A small incision or puncture was made in the scrotal skin, and a lateral-opening biopsy needle connected to a negative-pressure syringe was inserted percutaneously to aspirate testicular parenchymal tissue. Samples were processed for hematoxylin and eosin (H&E) staining, immunostaining of spermatogonia, or RNA analysis as required.

### 2.8 Letrozole treatment

Letrozole (Femara; Novartis Pharmaceuticals, Switzerland) was administered orally at 0.05 mg/kg body weight once daily. Treatment duration was 1 week for the treatment group and 3 months for the safety group, according to group assignment. No placebo was administered to control groups.

### 2.9 Semen collection, analysis, and fertility assessment

Semen samples were collected by manual stimulation into pre-warmed sterile containers. A mature teaser bitch in natural estrus was used consistently for each collection to maintain comparable sexual arousal and minimize collection-related variation. The sperm-rich second fraction of the ejaculate was collected. Ejaculate volume was measured immediately after collection. Sperm motility was evaluated by placing a drop of semen on a pre-warmed glass slide and analyzing it by computer-assisted sperm analysis (CASA). Progressive motility was the parameter used for statistical analysis; sperm concentration was recorded as sperm density. The presence of spermatozoa was evaluated under a light microscope.

Mating trials were performed only for the 1-week letrozole-treated group, the untreated aged azoospermic group, and the young fertile control group; the 3-month safety group was not mated. After spermatozoa were detected in the ejaculate and sperm quality was considered acceptable (progressive motility >50%), each male was mated with two healthy fertile females. To reduce inter-breed effects on litter size, females were matched to the male’s breed where a breed-matched female was available and, otherwise, were selected from breeds with similar normal litter sizes; the female breeds therefore paralleled the composition of the corresponding male groups (for example, Beagle, Golden Retriever, Japanese Akita, Border Collie, Shiba Inu, Welsh Corgi, Bichon Frise, and German Shepherd). Mating was performed every other day during the fertile window of estrus, determined by behavioral signs, serial vaginal cytology, and sequential serum progesterone monitoring. Female dogs were monitored for pregnancy after the final mating until delivery; pregnancy rate and litter size were recorded.

### 2.10 qRT-PCR

Seminiferous tubule biopsies were collected and immediately snap-frozen in liquid nitrogen and stored at −80 °C until processing. Total RNA was extracted from approximately 20–30 mg of frozen testicular tissue that had been homogenized in lysis buffer using a mechanical tissue homogenizer (QIAGEN, Germany), followed by purification with the MicroElute Total RNA Kit (Omega, United States) according to the manufacturer’s protocol. RNA concentration and purity were assessed using a spectrophotometer (NanoDrop, Thermo Fisher Scientific, United States), and RNA integrity was confirmed by agarose gel electrophoresis (clear 28S/18S ribosomal bands and absence of genomic DNA). Only samples with A260/A280 approximately 1.8–2.0 were used. Approximately 500 ng of total RNA per sample was reverse-transcribed into cDNA using oligo(dT) primers and the QuantiTect Reverse Transcription Kit (QIAGEN, Germany).

qRT-PCR was performed on an ABI StepOnePlus instrument (Applied Biosystems, United States) using a SYBR Green detection system (Takara, Japan) with 40 amplification cycles. Each assay included at least three biological samples, each analyzed in triplicate (*n* = 3 × 3). The PCR efficiency of each primer pair was determined by serial dilution of pooled cDNA to generate a standard curve with efficiencies ranged from 95 to 105%, and relative expression was calculated using the 2-ΔΔCt method. *GAPDH* was selected as the reference gene because it showed stable expression across the young fertile, aged azoospermic, and letrozole-treated groups in this study (comparable Ct values among groups) and is a widely validated housekeeping gene in canine testicular tissue. Primer sequences are listed in Table 2. Primers were designed with the NCBI Primer-BLAST tool. PCR conditions: initial denaturation at 95 °C for 2 min, followed by 40 cycles of 95 °C for 10 s and 60 °C for 30 s.

**Table 2:** Primer sequences used for qRT-PCR.

Gene (Accession)	Sequence (5′-3′)	Amplicon note
*GAPDH* (XM_038448971)	F: TCCATCTTCCAGGAGCGAGA/R: GGTTCACGCCCATCACAAAC	Reference gene
*IZUMO1* (XM_038656424)	F: ATCGATTGCGGAGTGCACGA/R: CACCATGGTCTTGGTCAGGG	Fertilization
*SPACA6* (XM_038656071)	F: GCCTTTAAGGGCCTCCTAGAC/R: TGCAGGCCTCGACTTCTTTA	Fertilization
*TMEM95* (XM_038665393)	F: CTGAGGGTTATGGCTTTGGCT/R: GGACTCCACCACGAGGC	Fertilization

### 2.11 Histological and immunohistochemical imaging

For histological evaluation, testicular samples were fixed in 4% paraformaldehyde (PFA) at 4 °C for 24 h, dehydrated through a graded ethanol series, cleared in xylene, and embedded in paraffin. Transverse sections 8 micrometer thick were cut and stained with H&E. Sections were examined and imaged using a bright-field light microscope (Olympus, Japan) with magnification of ×200/×400.

For immunofluorescence, fresh testicular samples were fixed in 4% PFA at 4 °C for 12 h, rinsed with PBS, and processed for whole-mount seminiferous tubule staining. Antigen retrieval and permeabilization were performed with proteinase K (20 μg/mL in PBS) at 37 °C for 30 min. Non-specific binding was blocked with 5% bovine serum albumin (BSA) in PBS containing 0.1% Triton X-100 at room temperature for 1 h. Sections were incubated overnight at 4 °C with primary antibody against *KIT* (clone D13A2, Cell Signaling Technology, United States; 1:100). After PBS washing, samples were incubated with Alexa Fluor 555-conjugated secondary IgG (Thermo Fisher Scientific, United States) at room temperature for 1 h in the dark. Nuclei were counterstained with Hoechst 33342 (10 μg/mL) for 10 min. Fluorescent images were acquired using a confocal laser scanning microscope (Leica TCS SP8, Germany) at standardized parameters (405 nm excitation for Hoechst 33342; 543 nm excitation for Alexa Fluor 555) and analyzed by Leica image analysis software.

### 2.12 Serum biochemistry analysis

Blood samples were collected from treatment and control dogs 24 h after the final letrozole dose (see section 2.6 for collection and serum preparation). Creatinine (CREA), alkaline phosphatase (ALP), and alanine aminotransferase (ALT) were measured using a Seamaty SMT-120VP biochemistry analyzer (Chengdu Seamaty Technology, China). The physiological reference range for serum CREA in healthy adult intact male dogs was 28–159 μmol/L; ALP was measured by the pNPP rate method (reference range 17–212 U/L); ALT was measured by the modified Reitman-Frankel rate method (reference range 5–125 U/L).

### 2.13 Seminal plasma analysis

Semen samples were centrifuged at 3000 × g (RCF) for 10 min to separate seminal plasma, which was then cleared of cellular debris by gentle filtration to improve assay accuracy. Zinc, fructose, and glucose were measured in the prepared seminal plasma. Zinc and fructose were measured using the automatic biochemistry analyzer (BEION T200, China). Glucose was measured using a fully automatic biochemistry analyzer (Mindray BS-2000 M, China).

### 2.14 Statistical analysis

All statistical analyses were performed using GraphPad Prism 9.0 (GraphPad Software, United States). Data are presented as mean +/− standard deviation (SD). For each comparison, normality was assessed using the Shapiro-Wilk test and homogeneity of variance using Levene’s test. Where these assumptions were met, parametric tests were used as the primary analyses: unpaired two-tailed Student’s *t*-test for two-group comparisons and one-way ANOVA followed by Tukey’s multiple-comparisons test for multi-group comparisons. Where assumptions were violated, non-parametric alternatives (Mann–Whitney U test for two-group comparisons and Kruskal-Wallis test followed by Dunn’s post-hoc test) were applied. Parametric tests were selected as the primary framework because the small, evenly sized groups in this exploratory study provide limited power for non-parametric procedures, and both approaches yielded consistent conclusions where compared. *p* < 0.05 was considered statistically significant and is marked as follows: * *p* < 0.05, ** *p* < 0.01, and *** *p* < 0.001. Raw data were recorded in laboratory notebooks and stored electronically in a repository.

## 3 Results

### 3.1 Impaired spermatogenesis and reduced testosterone in aged azoospermic dogs

Compared with young fertile dogs, aged azoospermic dogs showed profound reproductive dysfunction characterized by azoospermia (Figure 1), manifested by the absence of spermatozoa in the ejaculates. These dogs retained normal courtship and copulation behavior, and ejaculate volume was comparable to that of young fertile dogs (Figure 1C). Total testosterone concentrations were significantly lower in aged azoospermic dogs than in young fertile dogs (Figure 1D). Immunohistochemical analysis further showed disrupted spermatogonial differentiation in aged testes, indicated by loss of *KIT*-positive spermatogonia in seminiferous tubules (Figure 1E) (25). Elongating spermatids were also absent in aged testes (Figure 1F). These findings indicate that aged azoospermic dogs had impaired spermatogenesis and diminished sperm output, together with reduced serum testosterone that is indicative of disrupted reproductive endocrine homeostasis.

**Figure 1:**
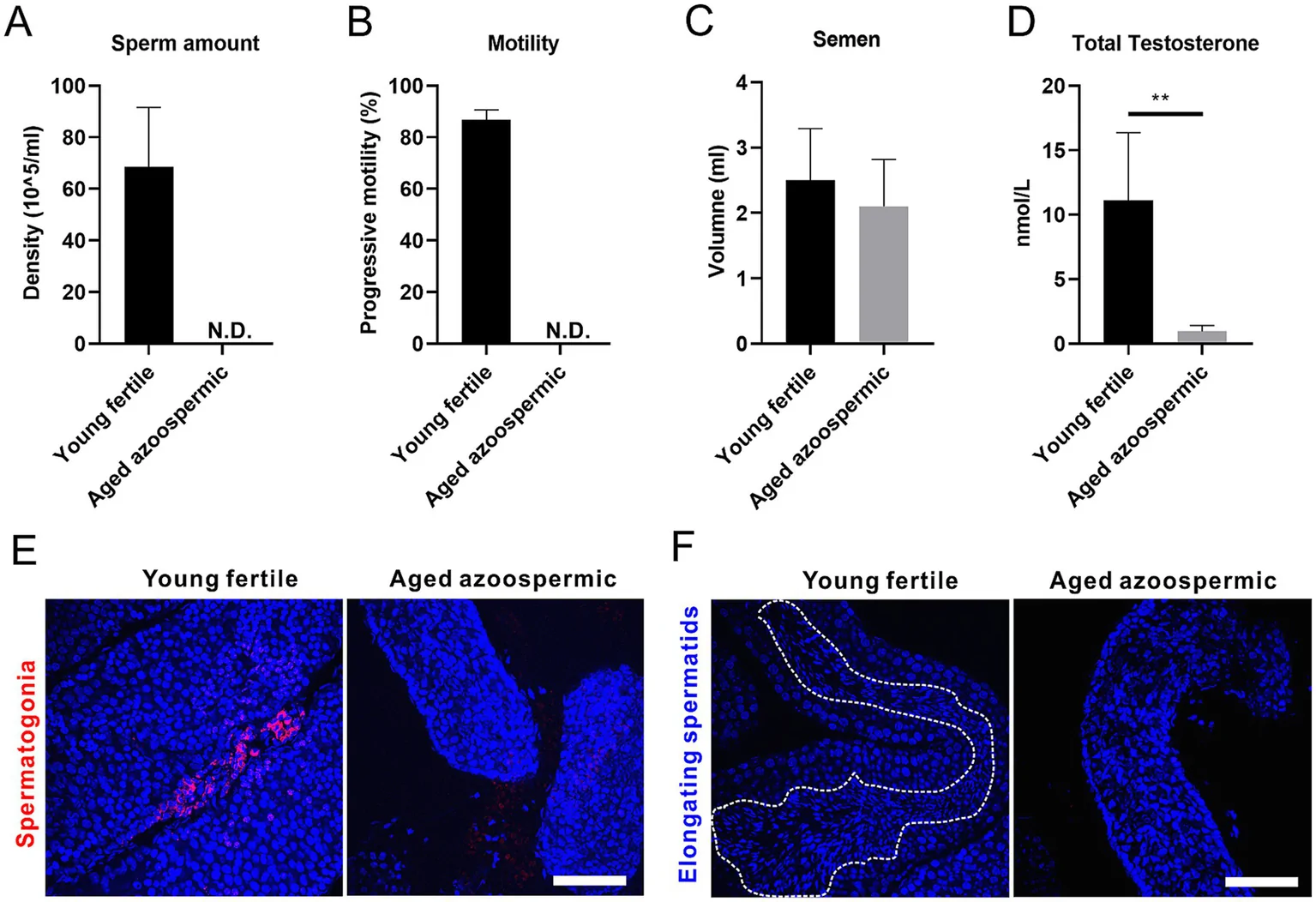
Impaired spermatogenesis and reduced testosterone in aged azoospermic dogs. Quantification of sperm amount **(A)**, progressive motility **(B)**, and semen volume **(C)** in young fertile and aged azoospermic dogs (*n* = 5). **(D)** Serum testosterone concentrations in the two groups. Data are mean ± SD; ** *p* < 0.01 vs. young fertile (unpaired *t*-test). N.D., not detected. **(E,F)** Immunofluorescence of *KIT*-positive spermatogonia **(E)** and elongating spermatids **(F)** in young fertile and aged azoospermic testes (*n* = 5). Scale bar, 100 μm.

### 3.2 Short-course letrozole treatment restores spermatogenesis and sperm-related profile

After 1 week of daily letrozole, serum testosterone increased and reached its maximum approximately 2 weeks after treatment initiation, then returned toward baseline by 4–5 weeks (Figure 2A). In the 1-week treatment group, spermatozoa reappeared in ejaculates approximately three months after treatment and remained detectable for up to 6 months, whereas spermatozoa remained absent throughout the follow-up in untreated aged azoospermic dogs (Figure 2B). Semen progressed from clear or pale-yellow fluid to normal milky-white semen, and total sperm number, total motility, and progressive motility improved relative to untreated aged azoospermic dogs and approached values observed in young fertile dogs (Figures 2B,C). Differentiating spermatogonia were detected from 1 month after treatment and persisted until at least 4 months after treatment (Figure 2D). Sperm motility was absent in untreated aged azoospermic dogs but gradually improved from 3 months onward, with recovery maintained from months 4 to 6 (Figure 2C). These results indicate that a 1-week letrozole course promoted testosterone recovery and sustained spermatogenesis in aged azoospermic dogs over the 6-month follow-up.

**Figure 2:**
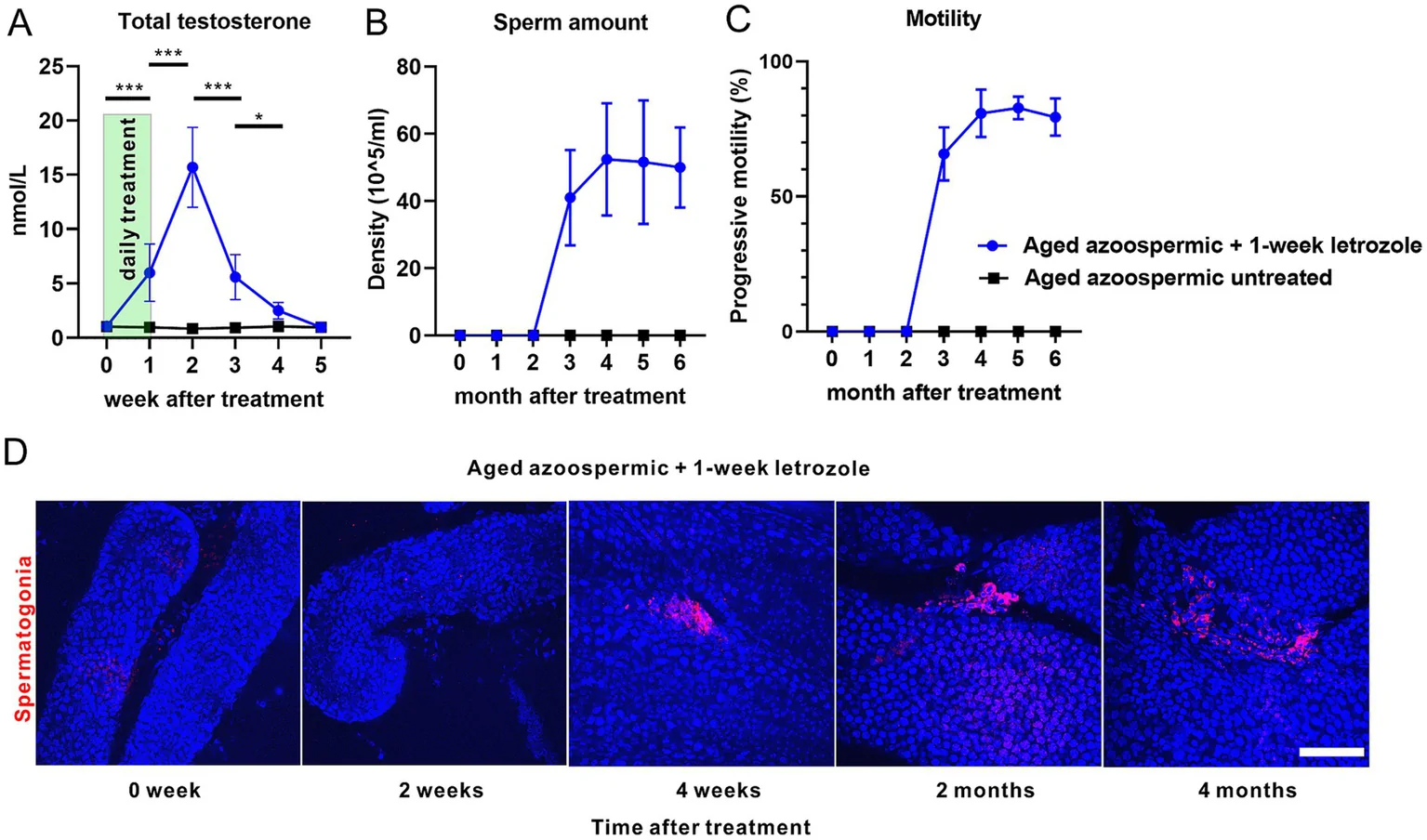
Short-course letrozole restores testosterone dynamics, spermatozoa output, sperm motility, and spermatogonial differentiation over 6 months. **(A)** Temporal testosterone profiles at 0–5 weeks in control and 1-week treatment groups, with young fertile reference. **(B,C)** Temporal sperm density **(B)** and progressive motility **(C)** at 0–6 months; young fertile and untreated aged azoospermic groups are shown for comparison (*n* = 3–5). Data are mean ± SD; * *p* < 0.05, *** *p* < 0.001 (one-way ANOVA, Tukey). **(D)** *KIT* immunofluorescence in aged dog testes at 0, 2, 4 weeks, 2 and 4 months after letrozole (*n* = 5). Scale bar, 100 μm.

### 3.3 Letrozole treatment improves fertility outcomes and seminiferous histological structure

Seminiferous tubules from treated dogs showed ongoing spermatogenesis 6 months after the 1-week treatment (Figure 3A). Untreated aged azoospermic dogs failed to sire offspring, whereas treated aged azoospermic dogs produced offspring after mating at 3 months post-treatment (Figure 3B). The mean litter size of treated dogs was lower than that of young fertile dogs (Figure 3B). These findings indicate that short-course letrozole restored testicular histology and fertility in aged azoospermic dogs, although litter size did not fully reach young fertile levels.

**Figure 3:**
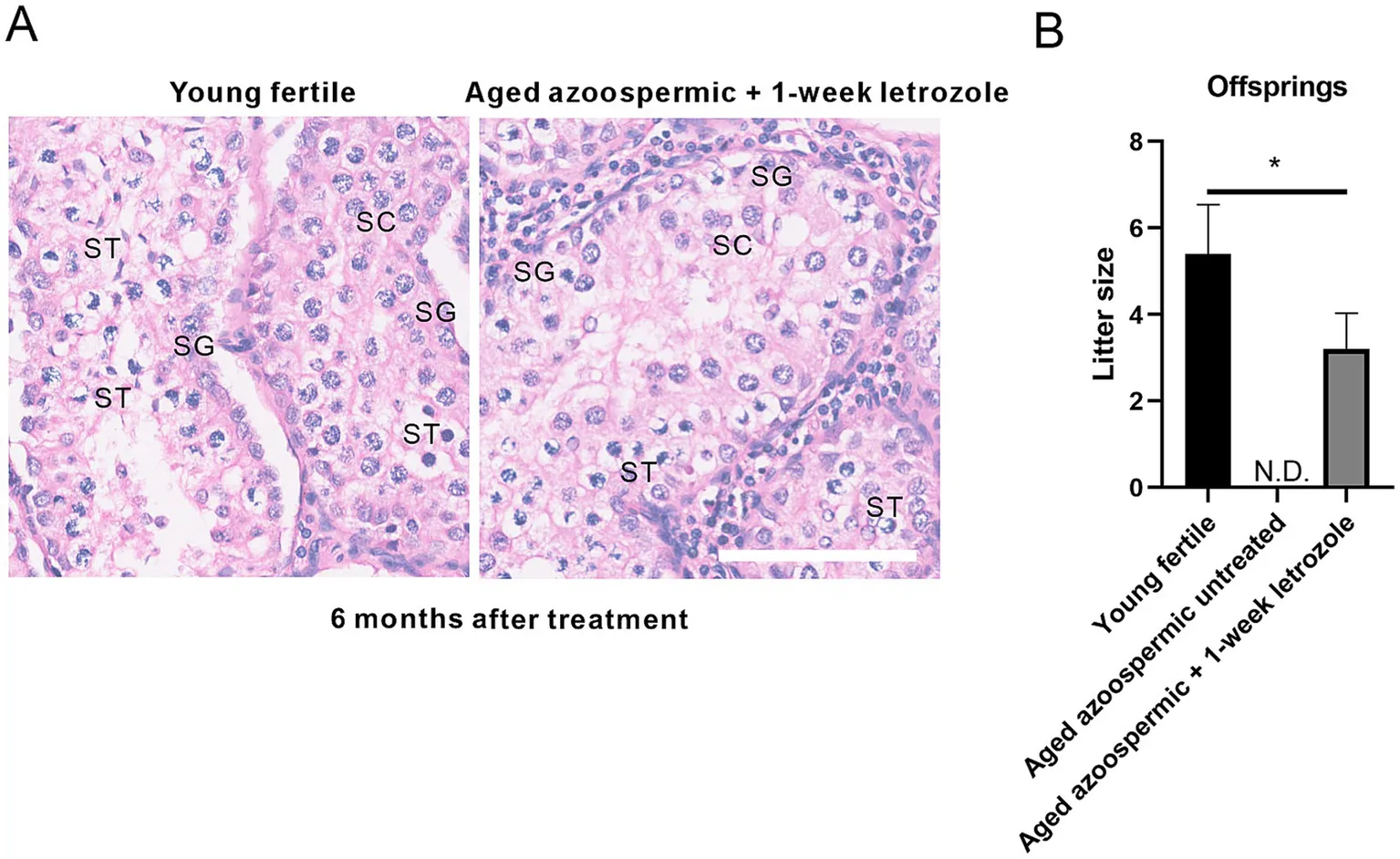
Letrozole treatment improves seminiferous histological structure and fertility outcomes. **(A)** Testicular H&E staining in young fertile, untreated aged azoospermic, and 1-week letrozole-treated dogs at 6 months post-treatment (*n* = 5). SG, spermatogonia; SC, spermatocytes; ST, spermatids. Scale bar, 100 μm. **(B)** Litter size in young fertile, untreated aged azoospermic, and 1-week letrozole-treated dogs. Data are mean ± SD; * *p* < 0.05, ** *p* < 0.01 (one-way ANOVA, Tukey).

### 3.4 Letrozole treatment improves gamete maturation and sperm-egg interaction gene expression

Testicular mRNA levels of *IZUMO1*, *SPACA6*, and *TMEM95*, which are required for sperm-egg fusion and fertilization (26), were reduced in aged azoospermic dogs and increased after letrozole treatment, although they remained lower than in young fertile dogs (Figure 4A). Seminal plasma zinc, fructose, and glucose, which support sperm viability and function, were measured at 3 months post-treatment. Fructose was reduced in untreated aged azoospermic dogs and partially restored after treatment; zinc and glucose showed similar directional changes (Figure 4B). These findings indicate that letrozole partially restored molecular and nutritional features associated with fertilization competence.

**Figure 4:**
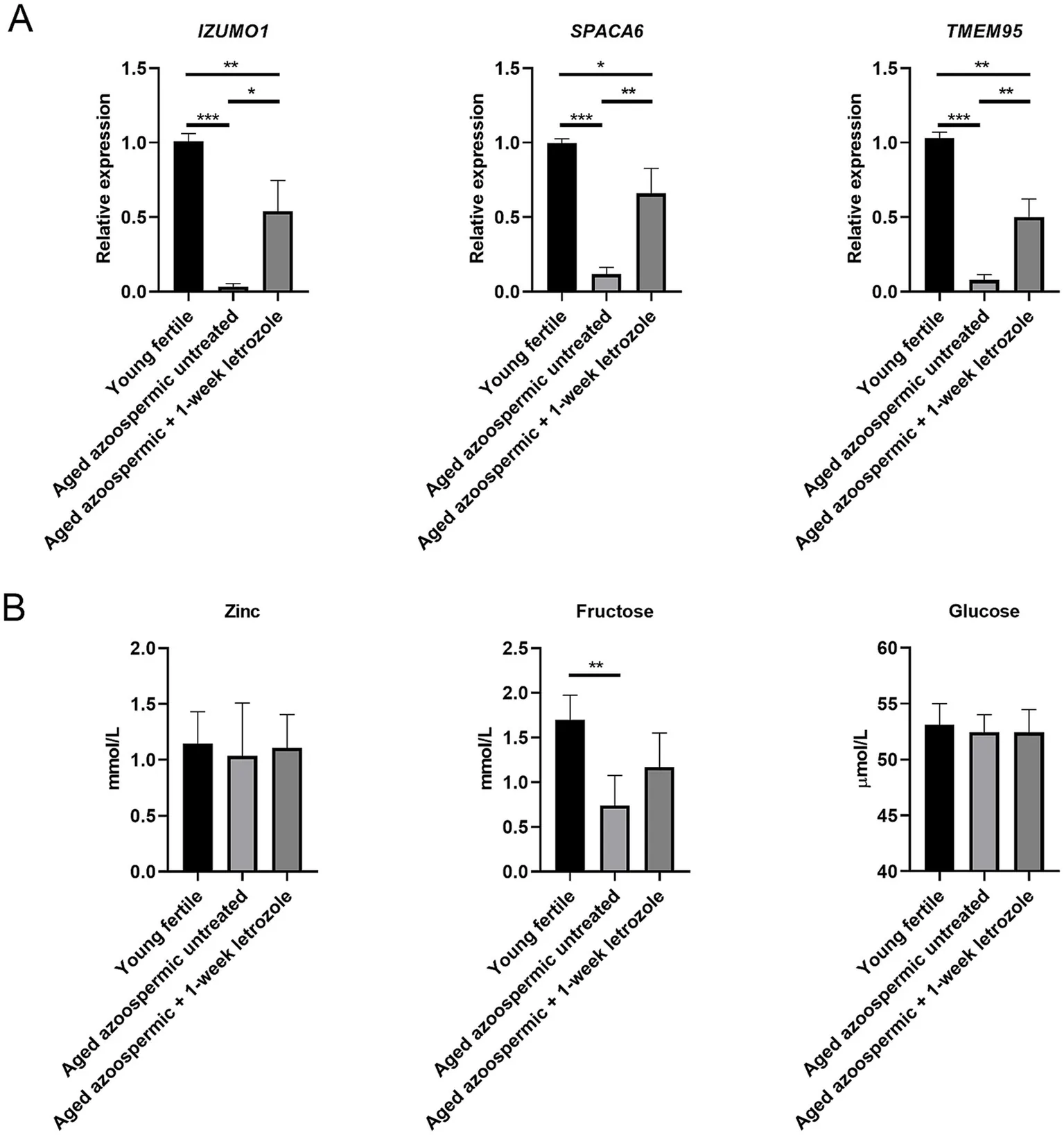
Letrozole treatment improves fertilization-related gene expression and seminal plasma profile. **(A)** Relative mRNA expression of *IZUMO1*, *SPACA6*, and *TMEM95* in young fertile, untreated aged azoospermic, and 1-week letrozole-treated dogs. **(B)** Seminal plasma zinc, fructose, and glucose concentrations in the three groups (*n* = 5). Data are mean ± SD; * *p* < 0.05, ** *p* < 0.01, *** *p* < 0.001 (one-way ANOVA, Tukey).

### 3.5 Impact of short and long-course letrozole treatment on target biochemical markers

Clinical monitoring revealed no obvious adverse events in letrozole-treated or control groups during the 6-month period. Serum ALT, a marker of hepatocellular injury, was not affected by letrozole treatment (Figure 5A). ALP was significantly higher in dogs receiving 3-month letrozole than in dogs receiving 1-week letrozole or in young fertile and untreated aged azoospermic dogs, in which ALP remained within the physiological range (Figure 5B). Creatinine, a marker of renal function, was comparable across groups after 1-week or 3-month treatment (Figure 5C). These data indicate that the 1-week regimen was not associated with detectable hepatic, renal, or ALP-related abnormalities, whereas longer treatment was associated with increased ALP.

**Figure 5:**
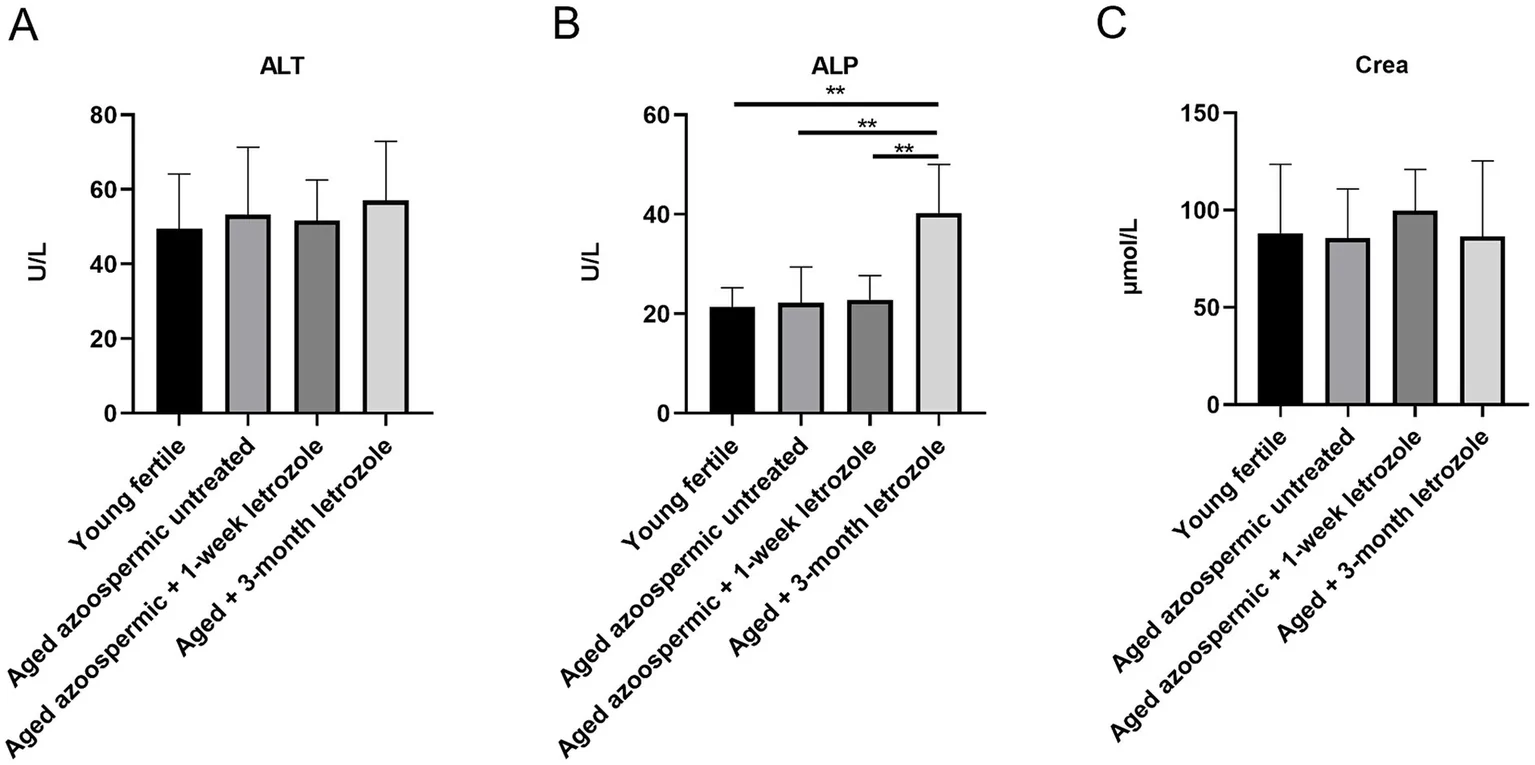
Serum biochemical indicators after 1-week and 3-month letrozole treatment in aged male dogs. **(A–C)** Serum ALT, ALP, and creatinine in young fertile, untreated aged azoospermic, 1-week letrozole, and 3-month letrozole groups (*n* = 3–5). Data are mean ± SD; ** *p* < 0.01 (one-way ANOVA, Tukey).

## 4 Discussion

The present study demonstrated that a 1-week course of letrozole restored spermatogenesis and fertility in aged azoospermic male dogs, accompanied by a transient increase in serum testosterone. This outcome is consistent with reports that aromatase inhibition improves spermatogenesis in aged roosters and bucks (10, 12), and extends those findings to a companion-animal model in which effective treatments are scarce. The recovery timeline is compatible with the duration of canine spermatogenesis, estimated at approximately 60–70 days (9, 16), and the early reappearance of differentiating spermatogonia within 2–4 weeks suggests that letrozole promotes renewal of the aged seminiferous epithelium, although the direct cellular mechanism was not evaluated in this study and letrozole does not act directly on germ cells. Mechanistically, aromatase inhibition is expected to reduce estrogenic negative feedback on the HPG axis, thereby increasing gonadotropin signaling and supporting testosterone production (6, 11, 13). The persistence of spermatogenesis after testosterone returned toward baseline indicates that the benefit depends on durable improvement of the spermatogenic microenvironment rather than on sustained hormone elevation alone. On this basis, the study concludes that transient aromatase inhibition can reinitiate fertility in aged azoospermic dogs.

Although fertility was largely restored, litter size remained below that of young fertile dogs. This pattern aligns with established age-related declines in canine semen quality, including reduced motility, lower density, abnormal morphology, and a higher proportion of midpiece defects (17–19). The incomplete recovery of fertilization-related transcripts (*IZUMO1*, *SPACA6*, *TMEM95*) and the only partial restoration of seminal fructose likely contributed to the smaller litters, because these factors support gamete interaction and sperm viability (20–22). The discrepancy between recovered sperm presence and submaximal fertilization competence probably reflects persistent age-related defects in post-meiotic maturation that a single short hormonal intervention cannot fully reverse. The study therefore interprets the restored fertility as partial rather than complete reproductive rejuvenation.

A further finding was that the 1-week regimen showed a favorable short-course safety profile relative to 3-month exposure, as reflected by the absence of detectable ALT, creatinine, or ALP alterations with short-course treatment. One week of treatment was not associated with detectable changes in ALT, creatinine, or ALP, whereas 3 months of treatment increased ALP, a marker that can reflect liver or bone metabolism. Because ALT remained unchanged, a hepatic source of the ALP rise appeared limited, although bone-specific ALP was not measured; creatinine is also relatively insensitive for early renal impairment. These observations are consistent with concerns that extended letrozole exposure may alter metabolism, bone health, and prostate parameters in dogs (6, 14), and they support the hypothesis that minimizing exposure reduces safety risk. The study concludes that the ultra-short course represents a lower-exposure strategy, while acknowledging that definitive safety requires more sensitive renal and bone markers.

From a clinical perspective, these results address a condition for which established treatments are limited (2, 23). A 1-week oral protocol that restores fertility could be valuable for breeding males and for owners wishing to preserve genetic lines in companion animals, provided that efficacy and safety are confirmed more broadly. The principal caveats are the small, breed-heterogeneous cohorts and the restricted set of safety markers, which preclude firm conclusions about breed-specific responses or long-term effects on the prostate and offspring. The study therefore recommends that future work define optimal dose and duration, evaluate breed-specific and larger populations, and incorporate sensitive endocrine, renal, and bone assessments as well as prostate imaging.

### 4.1 Limitations of the study

The present study has several limitations that constrain interpretation. First, the sample size was small—only five aged azoospermic dogs received letrozole and three served as untreated controls—reflecting the difficulty of recruiting this rare clinical population; larger studies are required to confirm efficacy and safety. Second, the groups comprised different breeds with varying body mass, adiposity, muscle content, and metabolic rate, and small dogs generally metabolize drugs faster than large breeds; purebred dogs may also carry breed-specific metabolic traits or predispositions (for example, to benign prostatic hyperplasia) that were not systematically evaluated, and prostate status was not assessed. These sources of biological variability, together with the limited panel of safety markers, mean that the observation that 1-week treatment was not associated with detectable changes in serum indicators of liver, kidney, or bone-related metabolism should be interpreted cautiously. Third, seminal plasma alkaline phosphatase was not measured, although histology argued against obstructive azoospermia. Fourth, only basal serum testosterone was measured without GnRH stimulation, limiting assessment of endocrine reserve given canine diurnal variation. Fifth, transcript expression may not reflect protein abundance in mature spermatozoa, and species-specific baseline data are lacking, as noted in livestock sperm transcriptome research (24). Sixth, endocrine profiling was limited to serum testosterone, and bone health assessment was restricted to serum ALP without vitamin D, calcium, parathyroid hormone, or magnesium. Future studies should evaluate long-term safety, including prostate, bone density, and offspring health, across breed-stratified and adequately powered cohorts.

## 5 Conclusion

In summary, a 1-week course of oral letrozole increased testosterone, restored spermatogenesis, and enabled pregnancies in aged azoospermic male dogs, with litter size remaining below young fertile levels and no detectable short-term biochemical toxicity. These findings support letrozole as a candidate low-exposure therapy for age-related canine azoospermia. Before clinical adoption, confirmatory studies with larger, breed-stratified cohorts, extended endocrine and safety profiling (including renal, bone, and prostate assessments), and defined dose–response relationships are recommended. This work provides a foundation for developing aromatase-inhibitor protocols in veterinary theriogenology.

## Data Availability

The original contributions presented in the study are included in the article/supplementary material, further inquiries can be directed to the corresponding author.
